# Oncogenic circ-SLC16A1 promotes progression of non-small cell lung cancer via regulation of the miR-1287-5p/profilin 2 axis

**DOI:** 10.1186/s11658-024-00549-x

**Published:** 2024-03-27

**Authors:** Mingming Jin, Tailei Yuan, Kaisai Tian, Jingjing Li, Qingqing Huang, Yongbin Chi, Gang Huang

**Affiliations:** 1 Shanghai Key Laboratory of Molecular Imaging, Jiading District Central Hospital Affiliated Shanghai University of Medicine and Health Sciences, Shanghai, 201318 People’s Republic of China; 2 Postgraduate Training Base of Shanghai Gongli Hospital, Ningxia Medical University, Shanghai, 200135 People’s Republic of China; 3 Department of Clinical Lab, Shanghai Pudong New Area Gongli Hospital, Shanghai, 200135 People’s Republic of China; 4 Jiangbei Hospital Affiliated to Xinglin College, Nantong University, Jiangsu, 210048 People’s Republic of China

**Keywords:** Circular RNA SLC16A1, Non-small cell lung cancer, microRNA-1287-5p, Profilin 2, Lymphatic metastasis

## Abstract

**Background:**

Circular RNAs (circRNAs) are single-stranded RNAs with covalently closed structures that have been implicated in cancer progression. However, the regulatory mechanisms remain largely unclear. So, the aim of this study was to reveal the role and regulatory mechanisms of circ-SLC16A1.

**Methods:**

In this study, next-generation sequencing was used to identify abnormally expressed circRNAs between cancerous and para-carcinoma tissues. Fluorescence in situ hybridization and quantitative reverse transcription polymerase chain reaction were performed to assess the expression patterns of circ-solute carrier family 16 member 1 (SLC16A1) in non-small cell lung cancer (NSCLC) cells and tissue specimens. The dual-luciferase reporter assay was utilized to identify downstream targets of circ-SLC16A1. Transwell migration, wound healing, 5-ethynyl-2′-deoxyuridine incorporation, cell counting, and colony formation assays were conducted to assess the proliferation and migration of NSCLC cells. A mouse tumor xenograft model was employed to determine the roles of circ-SLC16A1 in NSCLC progression and metastasis in vivo.

**Results:**

The results found that circ-SLC16A1 was upregulated in NSCLC cells and tissues. Downregulation of circ-SLC16A1 inhibited tumor growth by reducing proliferation, lung metastasis, and lymphatic metastasis of NSCLC cells, and arrested the cell cycle in the G1 phase. Also, silencing of circ-SLC16A1 promoted apoptosis of NSCLC cells. The results of bioinformatics analysis and the dual-luciferase reporter assay confirmed that microRNA (miR)-1287-5p and profilin 2 (PFN2) are downstream targets of circ-SLC16A1. PFN2 overexpression or circ-SLC16A1 inhibition restored proliferation and migration of NSCLC cells after silencing of circ-SLC16A1. PFN2 overexpression restored migration and proliferation of NSCLC cells post miR-1287-5p overexpression.

**Conclusions:**

Collectively, these findings show that miR-1287-5p/PFN2 signaling was associated with downregulation of circ-SLC16A1 and reduced invasion and proliferation of NSCLC cells. So, circ-SLC16A1 is identified as a mediator of multiple pro-oncogenic signaling pathways in NSCLC and can be targeted to suppress tumor progression.

## Introduction

Non-small cell lung cancer (NSCLC) is a common malignancy originating from the bronchial mucosa or glands [1–3]. Novel prognostic markers of lymphatic metastasis, distal metastasis, and chemotherapy resistance of NSCLC are needed to improve patient prognosis [4–6].

Circular RNAs (circRNAs), which are characterized by a closed ring structure with no 5′ cap or 3′ polyadenylated tail [7, 8], are important regulators of gene expression and differentially expressed in tumor cells and tissues [9]. Various circRNAs have been implicated in tumor progression, which is closely related to patient prognosis [10]. CircRNAs can form regulatory networks with microRNAs (miRNAs) that regulate specific genes associated with the biological processes of cancer cells [11]. Therefore, circRNA–miRNA networks present potential diagnostic and therapeutic markers of NSCLC. Further insight into the role and mechanism of circRNAs for NSCLC may contribute to understand the development and progression of NSCLC.

In the present study, we identify a circRNA derived from solute carrier family 16 member 1 (SLC16A1), hsa_circ_0013561 designated as circ-SLC16A1, by analyzing expression profiles of circRNAs in NSCLC. circ-SLC16A1 is frequently upregulated in NSCLC compared with matched adjacent nontumorous tissues. High expression of circ-SLC16A1 portends poor prognosis. The results found that abnormal expression of circ-SLC16A1 is associated with dysregulation of the miR-1287-5p/profilin 2 (PFN2) signaling pathway in NSCLC cells and promotes disease progression.

## Materials and methods

### Study approval

The study protocol was approved by the Institutional Animal Care and Use Committee of Shanghai University of Medicine and Health Sciences (approval no. 2021-GZR-18-340406198707142817) based on the Basel Declaration and conducted in accordance with the Guide for the Care and Use of Laboratory Animals (https://www.ncbi.nlm.nih.gov/books/NBK54050/).

### Animals

Nude BALB/c female mice (age, 4 weeks; mean body weight, 15–20 g) were obtained from the Shanghai Laboratory Animal Research Center (Shanghai, China) and housed in an animal care facility at constant temperature (22 °C) and relative humidity (55%) under a 12-h light:dark cycle with ad libitum access to food and water.

### Strand-specific library construction and next-generation sequencing (NGS)

Total RNA was isolated from NSCLC tissues using TRIzol reagent (Invitrogen Corporation, Carlsbad, CA, USA). Then, 3 μg of total RNA free of ribosomal and noncoding RNA were used for the preparation of transcriptome libraries for NGS with the VAHTS Total RNA-seq (H/M/R) Library Prep Kit for Illumina^®^ (Vazyme Biotech Co., Ltd, Nanjing, China). The purified RNA was treated with 40 U of RNase R (Epicenter Technologies Pvt., Ltd., Thane, India) at 37 °C for 3 h. An RNA-seq library was constructed using the KAPA Stranded RNA-Seq Library Prep kit (Hoffmann-La Roche AG, Basel, Switzerland) and sequenced with the HiSeq 4000 platform (Illumina, Inc., San Diego, CA, USA), which was conducted by Shanghai Aksomics Biotechnology Co., Ltd. (Shanghai, China). The study protocol was approved by the Committee of Shanghai University of Medicine and Health Sciences (approval no. 2023-GZR-18-340406198707142817) based on the Helsinki Declaration.

### Tissue chips and fluorescence in situ hybridization (FISH)

NSCLC tissue chip samples of 76 patients were obtained from Biotechwell (Shanghai, China). A digoxigenin (DIG)-labeled probe (DIG-5′-GCC ACC ACT TTT AGG CCA CTT TTA G-3′-DIG) for detection of circ-SLC16A1 was purchased from Guangzhou Jisai Biotechnology Co., Ltd. (Guangzhou, China). For FISH analyses, the tissue specimens were counterstained with the DNA-specific fluorescent dye 4′,6-diamidino-2-phenylindole (Shanghai Yeasen Biotechnology Co., Ltd., Shanghai, China) for 15 min to visualize the nuclei. The stained tissue specimens were imaged with a laser scanning confocal microscope (LSM 700; Carl Zeiss GmbH, Oberkochen, Germany).

### Cell culture

Human adenocarcinoma alveolar basal epithelial (A549, #STCC10201G, Servicebio, Wuhan, China) cells, human lung adenocarcinoma epithelial (HCC1833, #CBP60570, COBIOER BIOSCIENCES CO., LTD, Nanjing, China) cells, and normal (nontumorigenic) human lung epithelial (BEAS-2B, #STCC10202G, Servicebio, Wuhan, China) cells were obtained and cultured in Dulbecco’s modified Eagle’s medium (DMEM; Thermo Fisher Scientific, Waltham, MA, USA) supplemented with 10% fetal bovine serum (FBS; Thermo Fisher Scientific).

### RNase R treatment

The isolated RNA (2 μg) was digested with RNase R (0.2 μL; 20 U/μL) and 10 × RNase R reaction buffer (0.6 μL) at 37 °C for 30 min. Diethyl pyrocarbonate‐treated water (0.2 μL) was used as a control.

### RNA overexpression or interference

For the overexpression and knockdown experiments, the cells were transfected with an inhibitor of miR-1287-5p, circ-SLC16A1 silencing vector, or PFN2-overexpression vector.

### Cell migration assay

The cell migration assay was conducted using a 24-well transwell chamber (BD Biosciences, San Jose, CA, USA) with Hcc1833 and A549 cells in the upper chamber, and 500 µL of DMEM supplemented with 20% FBS in the lower chamber. The cells were cultured at 37 °C for 24 h. Afterward, the cells adhered to the bottom chamber were fixed with 4% paraformaldehyde for 30 min, stained with 0.1% Crystal Violet (Shanghai Yisheng Biotechnology, Shanghai, China), and imaged under an inverted microscope (Axio Observer D1; Carl Zeiss GmbH).

### Wound healing assay

The control and transduced cells (3.5 × 10^5^) were cultured in DMEM supplemented with 10% FBS in the wells of six-well plates. When the confluency reached 90–95%, a “scratch” was created using the tip of a 1-mL pipette and the detached cells were washed off with phosphate-buffered saline and then cultured in serum-free medium. At 0, 1, 2, and 3 days post-seeding, the wound area was imaged under a microscope. The wound area was calculated using ImageJ software (https://imagej.nih.gov/ij/). The cell scratch healing rate was calculated as (scratch area at 0 h − scratch area at timepoint)/(scratch area at 0 h) × 100%.

### Cell proliferation assay

Cell proliferation was assessed using the 5-ethynyl-2′-deoxyuridine (EdU) Assay Kit (Thermo Fisher Scientific). Briefly, Hcc1833 and A549 cells (1 × 10^5^) were seeded in the wells of six-well plates. After 2 days, the cells were incubated with EdU for 2 h and then fixed with 4% formaldehyde.

### Clone formation assays

To assess colony formation, the cells (2 × 10^3^/well) were cultured in DMEM supplemented with 10% (v/v) FBS in the wells of six-well plates. After 10 days, the formed colonies were fixed, stained, and photographed.

### Cell counting kit-8 (CCK-8) assay

Cell proliferation was also assessed using the CCK-8 assay. Briefly, Hcc1833 and A549 cells were incubated in culture medium supplemented with 10% CCK-8 solution at 37 °C for 0, 1, 2, and 3 days. The proliferation rate was determined by measuring the absorbance of the cell culture with a microplate reader.

### Quantitative reverse transcription polymerase chain reaction (RT-qPCR)

RNA was isolated from tumor tissues and cells using TRIzol reagent and reverse-transcribed into complementary DNA with the TaqMan™ MicroRNA Reverse Transcription Kit (Thermo Fisher Scientific). Fold changes in relative expression levels were determined with the 2^−ΔΔCT^ method. The following primers (forward/reverse) were used for RT-qPCR analysis: circ-SLC16A1: 5′-GAA TGC TGT CCT GTC CTC-3′/5′-TGC CAA TCA TGG TCA GAG-3′; miR-1287-5p: 5′-GGU AAC CAG UCC UUA A-3′/5′-AAG GUU CCA CGG GGG-3ʹ; *PFN2*: 5′-ATG ATT GTA GGA AAA GAC CGG GA-3′/5′-GCA GTC ACC ATC GAC GTA TAG AC-3′; U6: 5′-CTC GCT TCG GCA GCA CA-3′/5′-AAC GCT TCA CGA ATT TGC GT-3′; and glyceraldehyde 3-phosphate dehydrogenase (GAPDH): 5′-TGT GGG CAT CAA TGG ATT TGG-3′/5′-ACA CCA TGT ATT CCG GGT CAA T-3′.

### Dual-luciferase reporter assay

Putative miR-1287-5p-binding sites of the 3′-untranslated region (UTR) of *PFN2* and circ-SLC16A1 were cloned into the psi-CHECK vector (Promega Corporation, Madison, WI, USA). Firefly luciferase 3′-UTR or circ-SLC16A1 were used as primary luciferase signals. The clones were named *PFN2*-wild-type (WT)/circ-SLC16A1-WT and *PFN2*-MUT/circ-SLC16A1-MUT. The psi-CHECK vector containing the *Renilla* luciferase signal was used for normalization of the firefly reporter signal. Hcc1833 cells were transfected with the vectors using Lipofectamine™ 2000 Transfection Reagent (Thermo Fisher Scientific). Firefly and *Renilla* luciferase activities were measured 24 h post transfection.

### In vivo tumor xenograft model

The right flank of each mouse was injected with viable WT or sh-circ-SLC16A1-transfected Hcc1833 cells (2 × 10^6^). Tumor size was measured using Vernier calipers every 5 days for 1 month. The tumor size was calculated as width^2^ × length × 0.5. Relative Ki-67 expression in tumor samples was determined by immunohistochemical analysis.

Stably transfected luminescence-labeled Hcc1833 (Luc-Hcc1833) cells or transfected with a negative control (NC) were used to assess metastasis. The mice were injected with sh-circ-SLC16A1 via the tail vein. At 4 weeks post-injection, lung metastasis was confirmed with a bioluminescence imaging system. Metastatic foci of the lung tissues were stained with hematoxylin and eosin (H&E). Metastasis to the plantar lymphatic network was detected by staining for cluster of differentiation 31 (CD31).

### Western blot assay

Total protein was isolated from the cells and tissue samples with radioimmunoprecipitation assay buffer (Beyotime Institute of Biotechnology, Shanghai, China) and quantified using the bicinchoninic acid assay. The proteins (20 µg) were separated by electrophoresis with 10% sodium dodecyl sulfate polyacrylamide gels and electrotransferred to polyvinylidene fluoride membranes, which were incubated at 4 °C overnight with primary antibodies against PFN2 (Abcam, Cambridge, UK) and GAPDH (Abcam), followed by horseradish peroxidase-conjugated goat anti-rabbit secondary antibodies (Abcam). The protein bands were developed using chemiluminescence western blotting reagent (GE Healthcare Life Sciences, Chicago, IL, USA) and quantified with Quantity One software (Bio-Rad Laboratories, Hercules, CA, USA).

### Statistical analysis

The data are presented as mean ± standard deviation. Comparisons of groups were conducted with Prism software (GraphPad Software, Inc., San Diego, CA, USA). A probability (*P*) value ≤ 0.05 was considered statistically significant.

## Results

### Abnormal circ-SLC16A1 expression was associated with progression of NSCLC

**Fig. 1:**
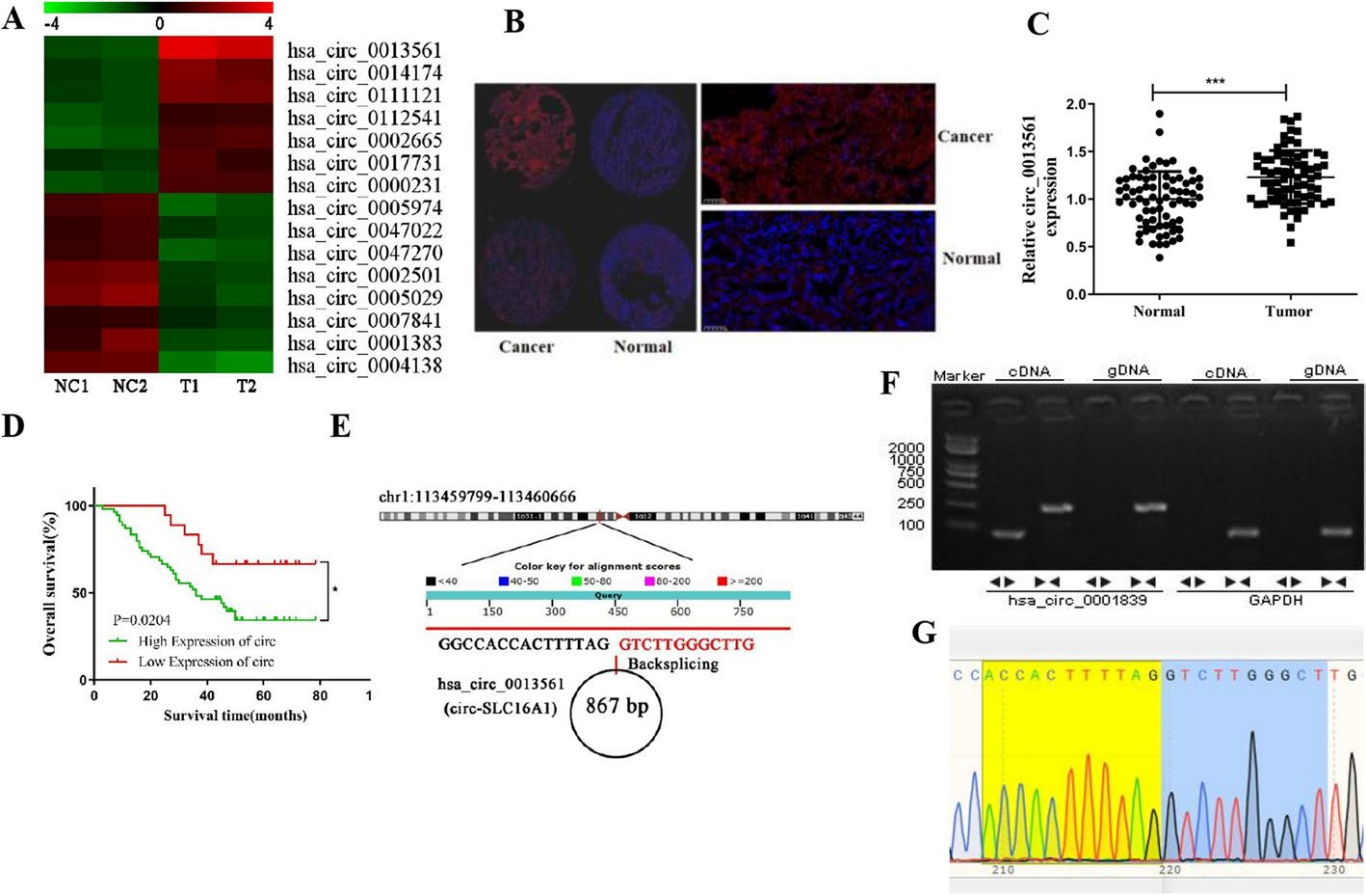
circ-SLC16A1 expression in NSCLC tissues was increased. **A** High-throughput sequencing showed abnormal expression of circRNA between NSCLC tissues and para-carcinoma tissue. **B** and **C** FISH detection showed the expression and subcellular localization of circ-SLC16A1. Data presented as mean ± SD. ***P < 0.001. **D** Kaplan–Meier survival analysis was performed to analyze the correlation of circ-SLC16A1 with NSCLC patient prognosis. Data shown as mean ± SD. *P < 0.05. **E** The information of circ-SLC16A1. **F** circ-SLC16A1 and GAPDH were amplified by cDNA and gDNA of H1833 cells with divergent primers and convergent primers, respectively. **G** Sanger sequencing of the RT-PCR products of circ-SLC16A1

The results of NGS confirmed that various circRNAs were abnormally expressed (Fig. 1A). The FISH data showed that hsa_circ_0013561 (circ-SLC16A1) expression was relatively increased in NSCLC tissue as compared with the para-carcinoma tissue and mainly located in cytoplasm (Fig. 1B, C). Kaplan–Meier survival analysis found that high circ-SLC16A1 expression was negatively correlated with survival (Fig. 1D) and positively correlated with lymphatic metastasis and tumor–node–metastasis (TNM) classification (Table 1). Bioinformatics analysis (http://www.circbase.org/cgi-bin/simplesearch.cgi) revealed that hsa_circ_0013561 (circ-SLC16A1) was located at chr1:113459799–113460666 and included an 867-bp exon of *SLC16A1* (Fig. 1E). Agarose gel electrophoresis showed that circ-SLC16A1 was resistant to digestion by RNase R exonuclease, thereby confirming a circular structure (Fig. 1F). A putative circ-SLC16A1 back-spliced junction fragment was confirmed by RT-qPCR amplification with the primers used to amplify the complementary DNA, but not from genomic DNA, as verified by Sanger sequencing (Fig. 1G), suggesting that abnormal expression of circ-SLC16A1 is associated with progression of NSCLC.

**Table 1:** Correlation between clinicopathological features of NSCLC and expression of circSLC16A1 (*n* = 72)

Characteristic	Sample	circSLC16A1 expression		*P*-value
		Low expression (*n* = 18)	High expression (*n* = 54)	
Age		58.78 (9.82)	59.33 (11.40)	0.867
Gender				
Male	29	7	22	0.890
Female	43	11	32	
Lymphatic metastasis				
No	47	14	33	0.017
Yes	25	4	21	
TNM				
I–II	37	13	24	0.041
III–IV	35	5	30	

### Downregulation of circ-SLC16A1 inhibited tumor growth and proliferation of NSCLC cells

**Fig. 2:**
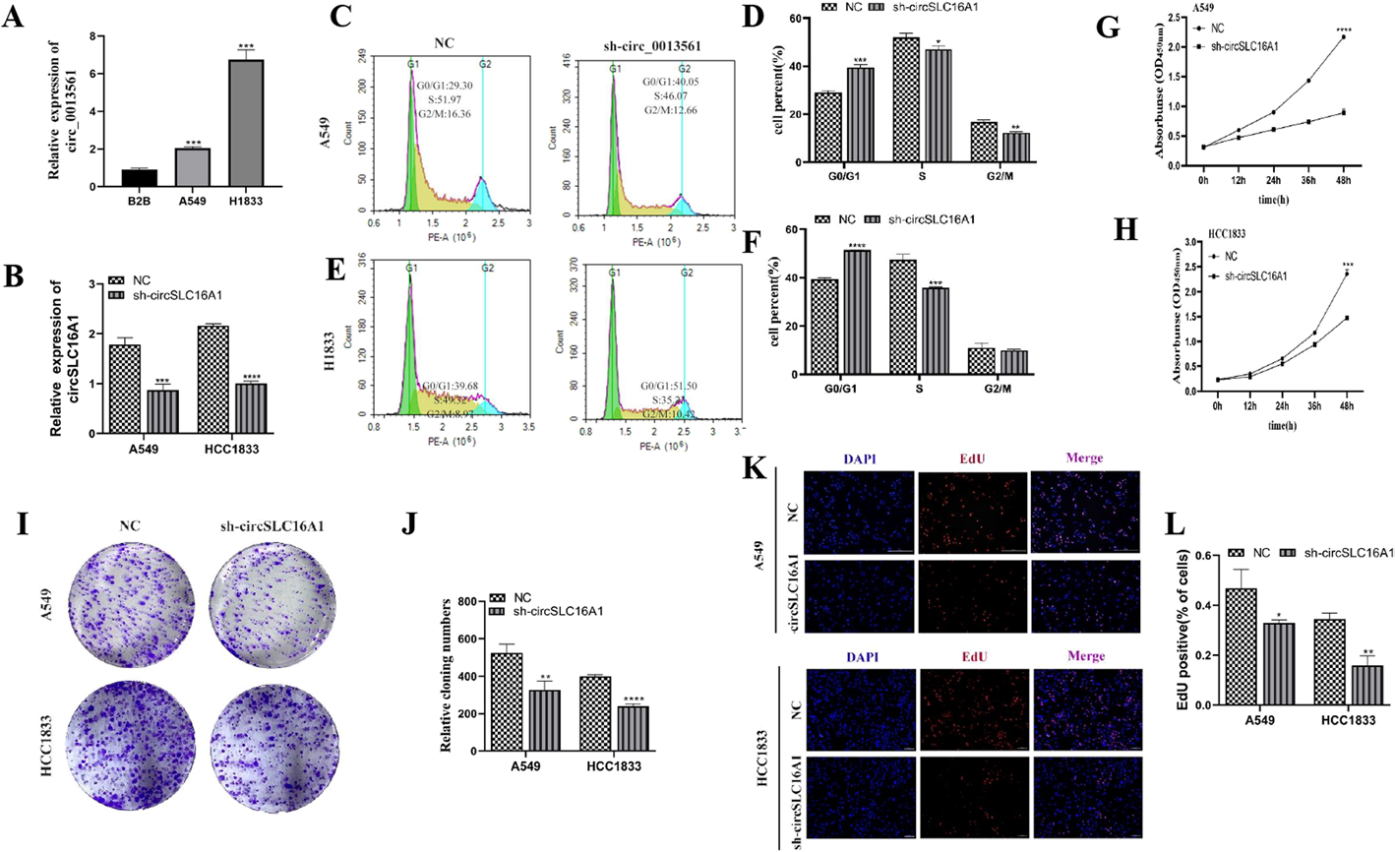
Downregulation of circ-SLC16A1 inhibited the proliferation ability of NSCLC cells. **A** RT-qPCR detection showed the expression of circ-SLC16A1 in BEAS-2B, A549, and H1833 cells. Data shown as mean ± SD. ***P < 0.001 versus BEAS-2B. **B** RT-qPCR detection showed the expression of circ-SLC16A1 in both A549 and H1833 cells after transfection with siRNA against circ-SLC16A1. **C**–**F** Flow cytometry for cell cycle detection. Data shown as mean ± SD. *P < 0.05, **P < 0.01, ***P < 0.001 versus NC. **G** and **H** CCK8 detection showed the proliferation ability of both A549 and H1833 cells. Data shown as mean ± SD. ***P < 0.001 versus NC. **I** and **J** Clone formation showed the proliferation ability in both A549 and H1833 cells. Data shown as mean ± SD. **P < 0.01, ***P < 0.001 versus NC. **K** and **L** EdU detection showed the proliferation ability in both A549 and H1833 cells. Data shown as mean ± SD. *P < 0.05, **P < 0.01 versus NC

The RT-qPCR results showed that circ-SLC16A1 expression was increased in A549 and H1833 cells as compared with BEAS-2B cells (Fig. 2A). Transfection with small interfering RNA (siRNA) significantly decreased expression of circ-SLC16A1 in both A549 and H1833 cells (Fig. 2B). Flow cytometry revealed that downregulation of circ-SLC16A1 maintained cells in the G1 phase (Fig. 2C–F). The results of the cell counting (Fig. 2G, H), clone formation (Fig. 2I, J), and EdU (Fig. 2K, L) assays showed that silencing of circ-SLC16A1 significantly inhibited cell proliferation.

**Fig. 3:**
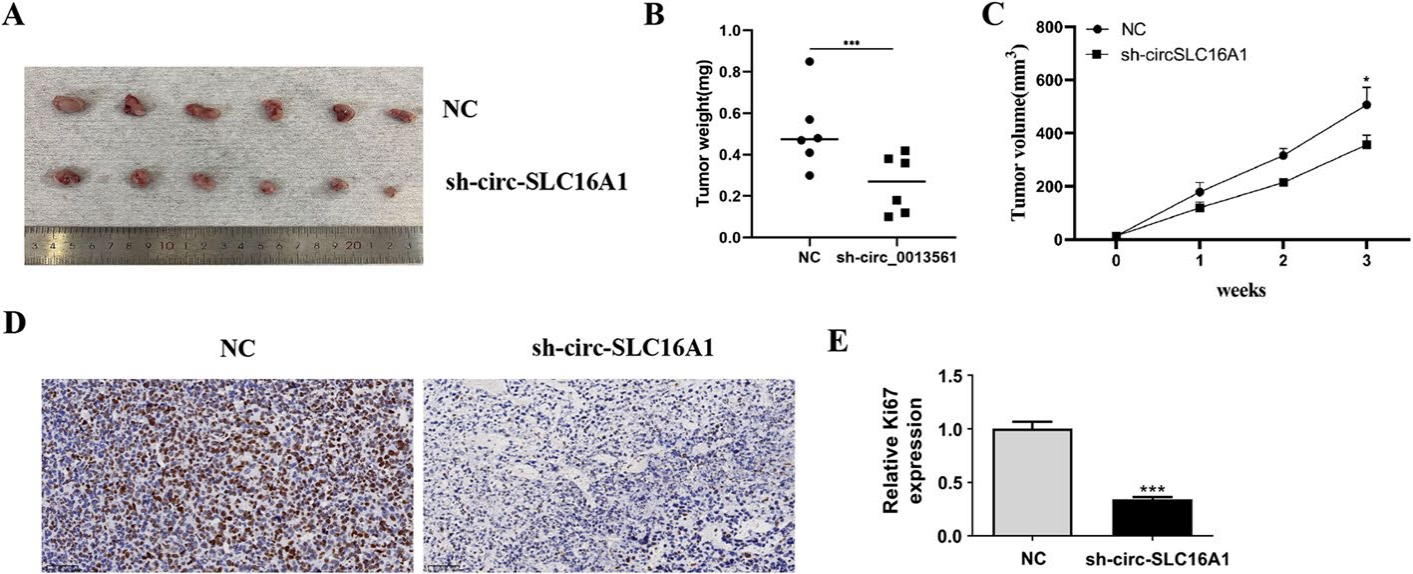
Downregulation of circ-SLC16A1 suppressed NSCLC tumor growth in vivo. **A** Representative images of H1833 tumor formation in xenografts from nude mice. **B** and **C** Summary of tumor volumes and weights in mice. Data presented as mean ± SD. *P < 0.05, ***P < 0.001 versus sh-NC. **D** and **E** Immunohistochemical staining showing the calculated percentage of Ki-67-positive cells and relative Ki-67-positive cells. Data presented as mean ± SD. ***P < 0.001 versus NC

H1833 cells were transfected with a lentivirus carrying circ-SLC16A1 to assess tumor formation. Tumor size was measured 5 days post grafting with Vernier calipers. Knockdown of circ-SLC16A1 reduced the size and weight of the xenograft (Fig. 3A–C). Immunohistochemical staining showed that decreased Ki67 expression was associated with reduced tumor growth via downregulation of circ-SLC16A1 (Fig. 3D, E).

### Downregulation of circ-SLC16A1 suppressed migration of NSCLC cells and promoted apoptosis

**Fig. 4:**
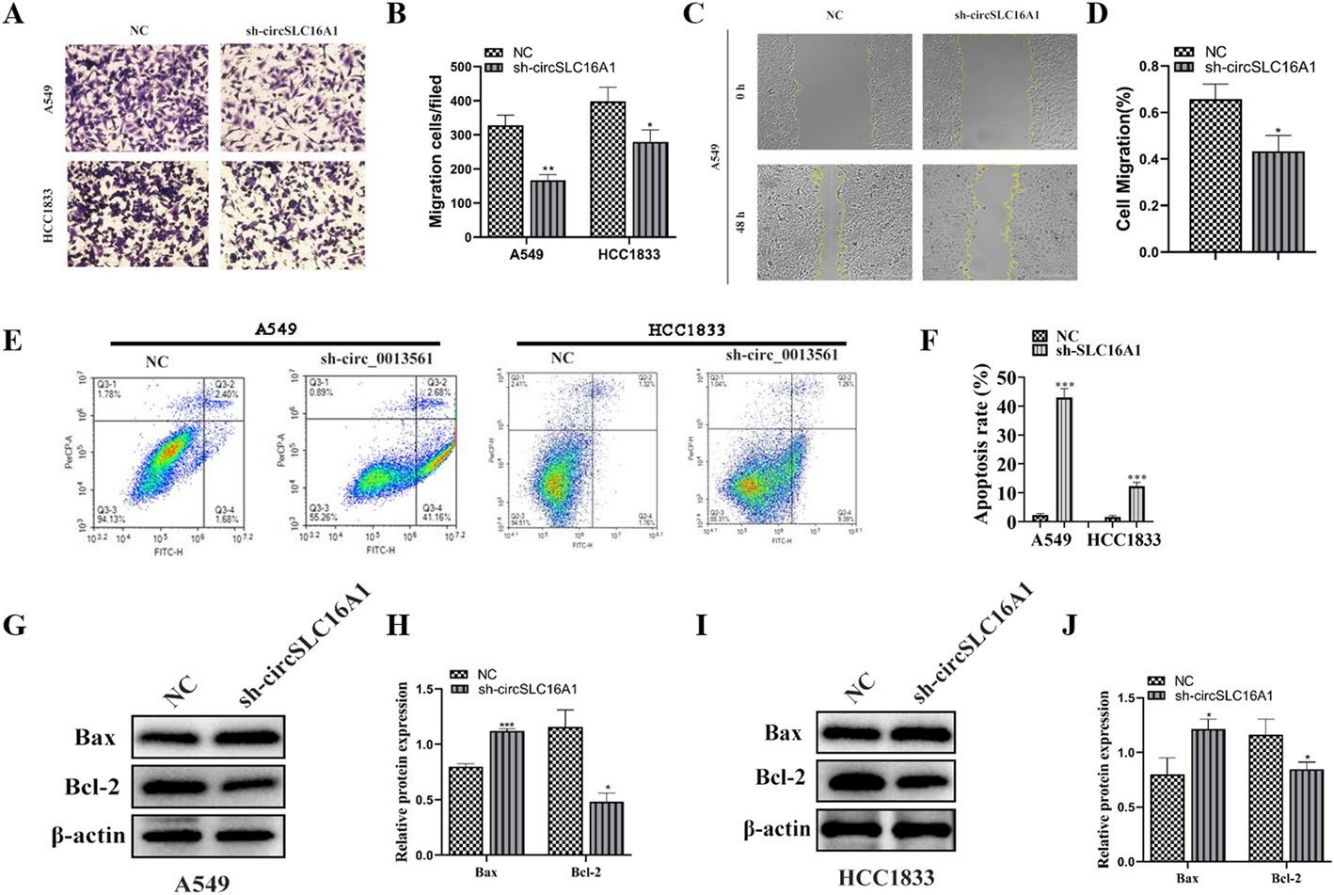
Downregulation of circ-SLC16A1 suppressed NSCLC cell migration and promoted cell apoptosis. **A** and **B** Transwell detection showing the migration of both A549 and H1833 cells after transfection with NC or si-circ-SLC16A1. Data presented as mean ± SD. *P < 0.05, **P < 0.01 versus NC. **C** and **D** Wound healing detection show the migration ability of A549. Data presented as mean ± SD. *P < 0.05 versus NC. **E** and **F** The apoptosis of A549 and HCC1833 cells was assessed using flow cytometry with annexin V-FITC staining. Data presented as mean ± SD. ***P < 0.001 versus NC. **G**–**J** Western blot detection showing the expression of apoptosis-related protein Bax and Bcl-2. Data presented as mean ± SD. *P < 0.05, ***P < 0.001 versus NC

**Fig. 5:**
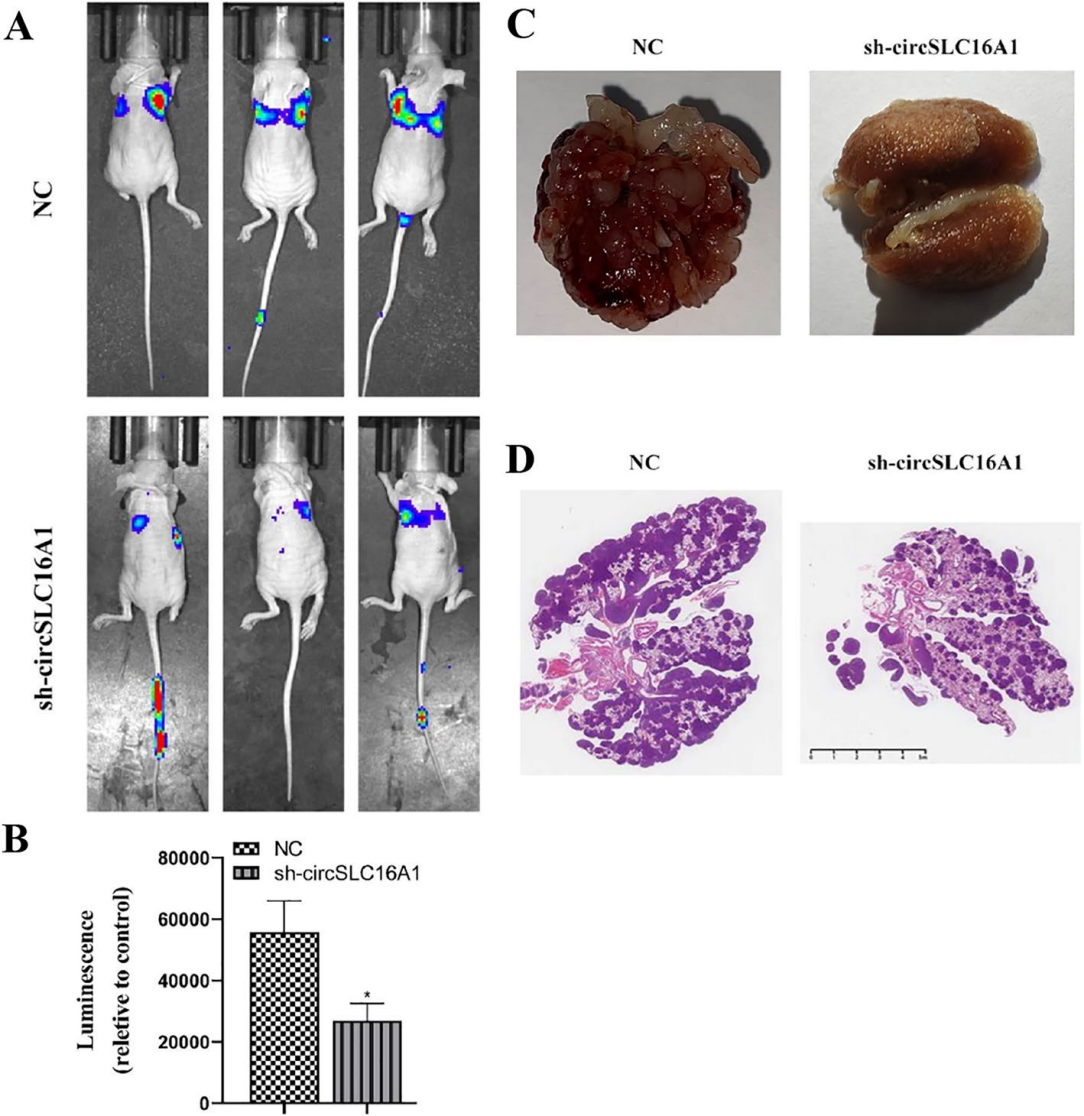
Downregulation of circ-SLC16A1 suppressed NSCLC metastasis. **A** and **B** Live image detection showing luc-H1833 cell pulmonary metastasis. Data presented as mean ± SD. *P < 0.05 versus NC. **C** Representative images of lung tissues. **D** The numbers of metastatic foci in lung tissues were calculated according to the H&E staining

**Fig. 6:**
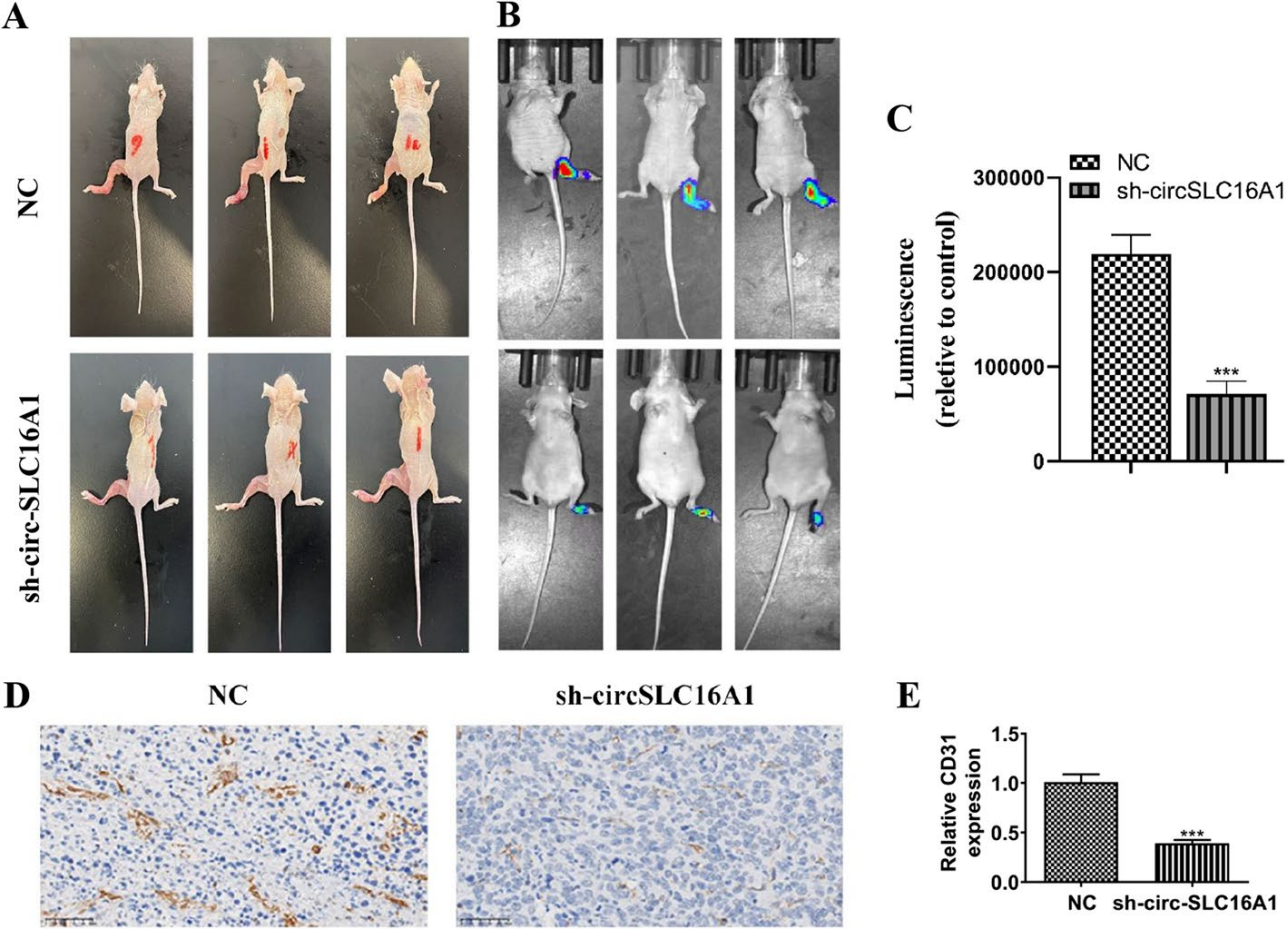
Downregulation of circ-SLC16A1 suppressed NSCLC lymphatic metastasis. **A**–**C** Live image detection showing luc-H1833 cell plantar lymph metastases. Data presented as mean ± SD. ***P < 0.001 versus NC. **D** and **E** Immunohistochemical detection showing the CD31 expression. Data presented as mean ± SD. ***P < 0.001 versus NC

The results of the transwell assay showed that downregulation of circ-SLC16A1 inhibited migration of H1833 and A549 cells (Fig. 4A, B). The wound healing assay revealed that silencing of circ-SLC16A1 inhibited migration of A549 cells (Fig. 4C, D). Flow cytometry showed that silencing of circ-SLC16A1 promoted apoptosis (Fig. 4E, F). Western blot analysis indicated that silencing of circ-SLC16A1 upregulated expression of Bax, but inhibited expression of Bcl-2 in H1833 and A549 cells (Fig. 4G–J). Living imaging revealed that silencing of circ-SLC16A1 decreased metastasis of luc-H1833 cells, as evidenced by decreased fluorescence (Fig. 5A, B). H&E staining confirmed that silencing of circ-SLC16A1 reduced the number of metastatic foci in lung tissues (Fig. 5C, D). Collectively, these results confirmed that downregulation of circ-SLC16A1 suppressed the invasive capacity of NSCLC cells. Live imaging showed that downregulation of circ-SLC16A1 inhibited plantar lymph metastases by luc-H1833 cells (Fig. 6A–C). Immunohistochemical analyses revealed that CD31 expression was decreased in plantar lymphoid tissue (Fig. 6D, E), suggesting that downregulation of circ-SLC16A1 inhibited lymph metastasis by NSCLC cells.

### PFN2 and miR-1287-5p were identified as downstream targets of circ-SLC16A1

**Fig. 7:**
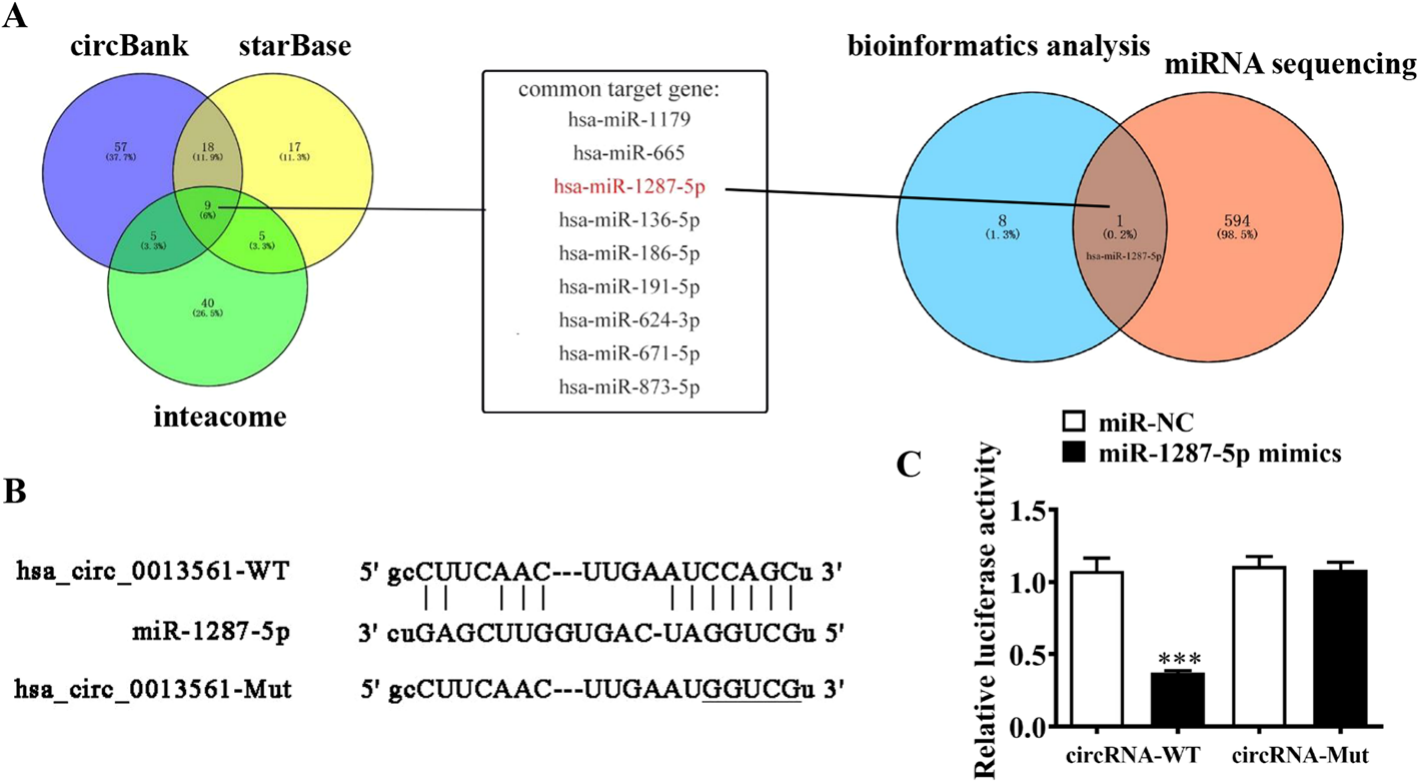
miR-1287-5p was the downstream target for circ-SLC16A1. **A** Bioinformatics and miRNA sequencing detection showing the downstream target of circ-SLC16A1. **B** Prediction of binding sites of miR-1287-5p in circ-SLC16A1. The MUT version of circ-SLC16A1 is presented. **C** Relative luciferase activity determined 48 h after transfection of H1833 cells with miR-1287-5p mimic/NC or circ-SLC16A1 WT/Mut. Data presented as mean ± SD. ***P < 0.001

Bioinformatics analysis was conducted in reference to the circBank (http://www.circbank.cn/), starBase (http://starbase.sysu.edu.cn/), and interactome (https://circinteractome.nia.nih.gov/) databases. The results showed that circ-SLC16A1 potentially interacts with nine miRNAs. Subsequent comparisons of the nine miRNAs with miRNA sequences of H1833 cells transfected with si-circ-SLC16A1 or a NC revealed that only miR-1287-5p was an actual downstream target of circ-SLC16A1 (Fig. 7A). The results of the luciferase reporter assay further confirmed that miR-1287-5p suppressed luciferase activity in WT cells (Fig. 7B, C), suggesting that miR-1287-5p is a downstream target of circ-SLC16A1.

**Fig. 8:**
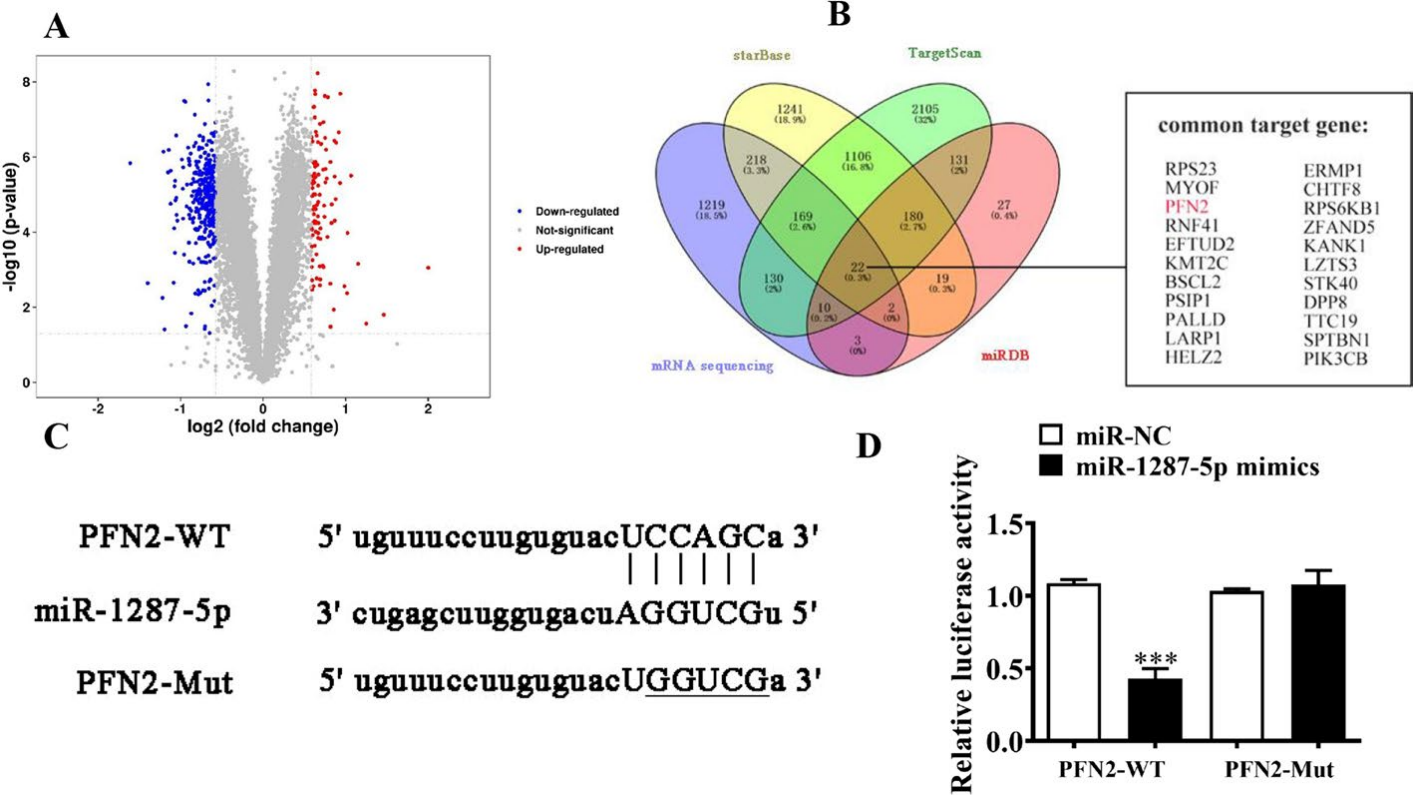
PFN2 was the downstream targets for miR-1287-5p. **A** Volcano plot showing the different expression mRNA. **B** Bioinformatics and mRNA sequencing detection showing the downstream target of miR-1287-5p. **C** Prediction of binding sites of miR-1287-5p and 3′-UTR of PFN2. The MUT version of 3′-UTR of PFN2 is presented. **D** Relative luciferase activity determined 48 h after transfection of H1833 cells with miR-1287-5p mimic/NC or 3′-UTR of PFN2 WT/Mut. Data presented as mean ± SD. ***P < 0.001

Various mRNAs were abnormally expressed in H1833 cells transfected with sh-circ-SLC16A1 as compared with those transfected with a NC (Fig. 8A). Bioinformatics analysis with the circBank, starBase, miRDB (http://www.mirdb.org/), and TargetScan (https://www.targetscan.org/vert_80/) websites found that PFN2 was a downstream target of miR-1287-5p (Fig. 8B). The luciferase reporter assay verified that miR-1287-5p suppressed luciferase activity in WT cells (Fig. 8C, D), suggesting that miR-1287-5p interacted with the 3′-UTR of PFN2 and confirming that PFN2 is a downstream target of miR-1287-5p.

### PFN2 overexpression and miR-1287-5p suppression both reversed inhibition of migration and proliferation of NSCLC cells induced by circ-SLC16A1 silencing

**Fig. 9:**
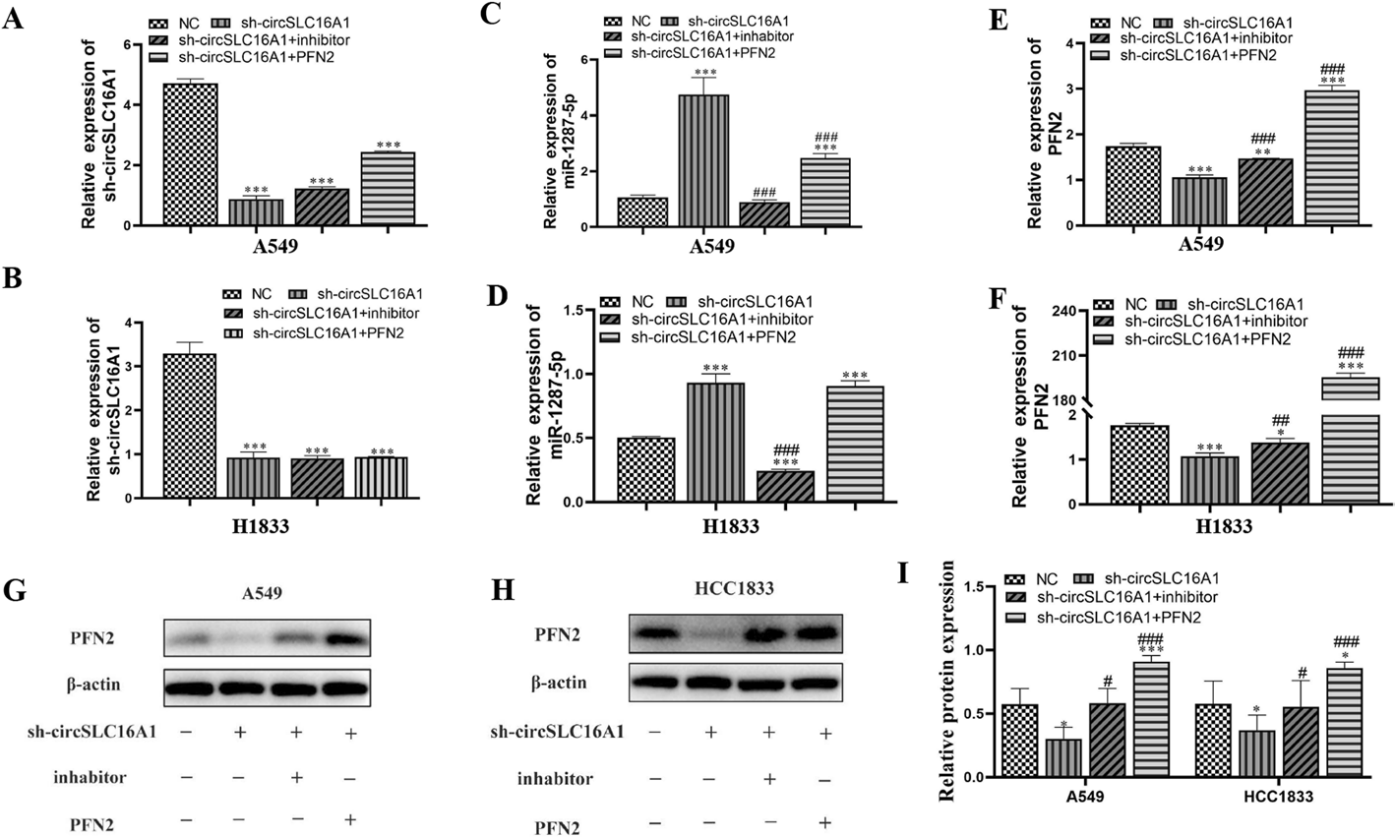
The regulation relationship among circ-SLC16A1, miR-1287-5p, PFN2. **A**–**F** RT-qPCR detection showing the expression of circ-SLC16A1, miR-1287-5p, and PFN2 in both A549 and H1833. Data presented as mean ± SD. *P < 0.05, **P < 0.01, ***P < 0.001 versus NC. ^##^*P* < 0.01, ^###^*P* < 0.001 versus sh-circ-SLC16A1. **G**–**I** Western blot detection showing the expression of PFN2 in both A549 and H1833. Data presented as mean ± SD. *P < 0.05, ***P < 0.001 versus NC. ^#^*P* < 0.05, ^###^*P* < 0.001 versus sh-circ-SLC16A1

The results of RT-qPCR analysis verified that siRNA-induced silencing decreased expression of circ-SLC16A1. However, treatment with an inhibitor of miR-1287-5p or overexpression of PFN2 had no effect on circ-SLC16A1 expression in H1833 and A549 cells (Fig. 9A, B), suggesting that miR-1287-5p and PFN2 are downstream targets of circ-SLC16A1. In addition, silencing of circ-SLC16A1 increased expression of miR-1287-5p, while overexpression of PFN2 had no effect on sh-circ-SLC16A1-induced expression of miR-1287-5p (Fig. 9B, C), suggesting that miR-1287-5p is located downstream of circ-SLC16A1. Also, silencing of circ-SLC16A1 reduced PFN2 expression, while downregulation of miR-1287-5p reversed this effect. Following transfection with the PFN2-overexpression vector, PFN2 expression was significantly increased at both the mRNA and protein levels (Fig. 9E–I), suggesting that circ-SLC16A1 promoted PFN2 expression by sponging miR-1287-5p.

**Fig. 10:**
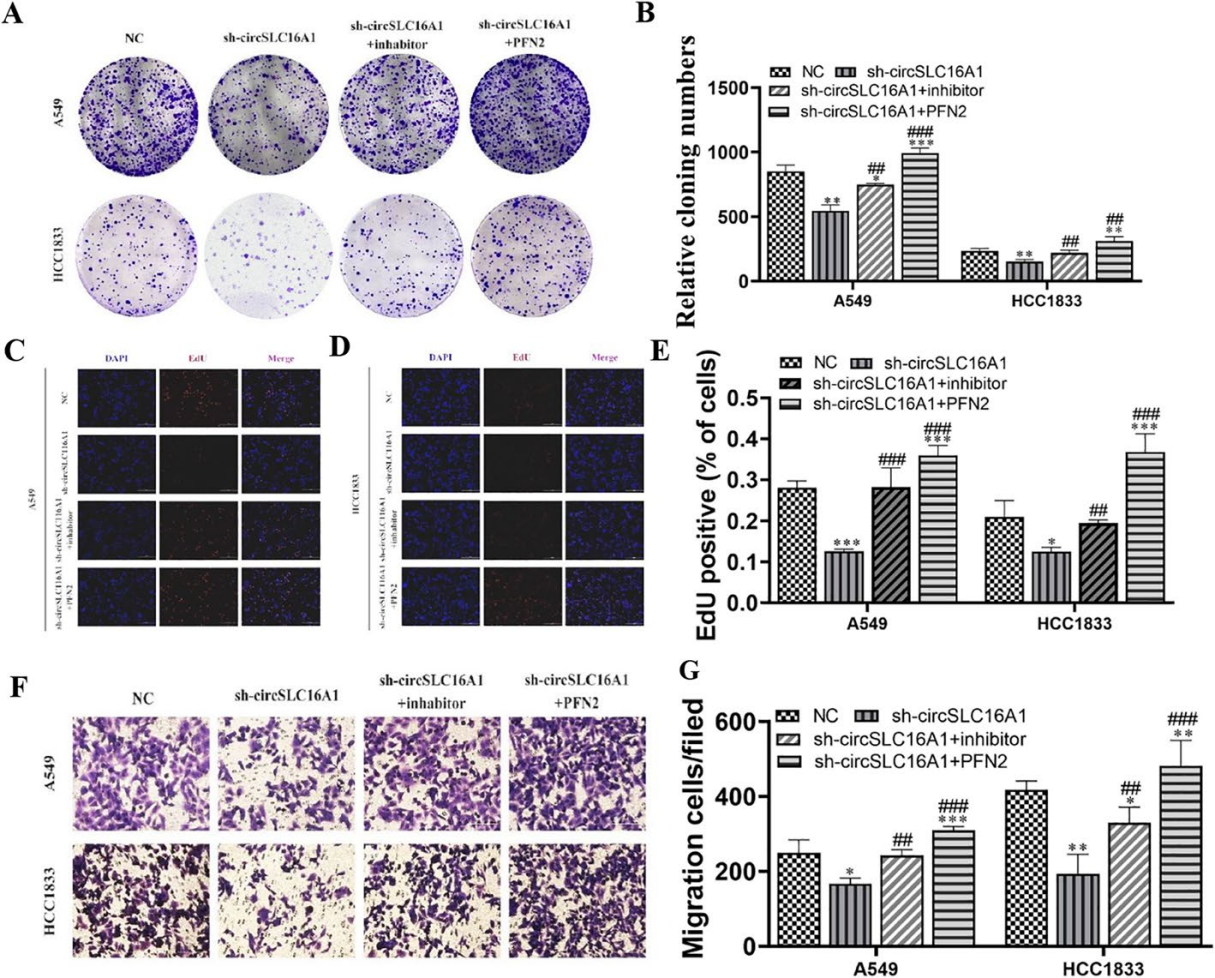
Overexpression of PFN2 or inhibition of miR-1287-5p reversed NSCLC cell proliferation and migration after silencing circ-SLC16A1. **A**, **B** Clone formation showing the proliferation ability in both A549 and H1833 cells. Data shown as mean ± SD. *P < 0.05, **P < 0.01, ***P < 0.001 versus NC. ^##^*P* < 0.01, ^###^*P* < 0.001 versus sh-circ-SLC16A1. **C**–**E** EdU detection showing the proliferation ability in both A549 and H1833 cells. Data shown as mean ± SD. *P < 0.05, ***P < 0.001 versus NC. ^##^*P* < 0.01, ^###^*P* < 0.001 versus sh-circ-SLC16A1. **F**, **G** Transwell detection showing invasion and migration of A549 and H1833 cells. Data shown as mean ± SD. *P < 0.05, **P < 0.01, ***P < 0.001 versus NC. ^##^*P* < 0.01, ^###^*P* < 0.001 versus sh-circ-SLC16A1

The results of the clone formation (Fig. 10A, B) and EdU (Fig. 10C–E) assays showed that PFN2 overexpression or miR-1287-5p inhibition reversed proliferation of H1833 and A549 cells after silencing of circ-SLC16A1. The results of the transwell assay revealed that PFN2 overexpression or miR-1287-5p inhibition reversed migration of H1833 and A549 cells after silencing of circ-SLC16A1 (Fig. 10F, G).

### Overexpression of PFN2 reversed NSCLC cell migration and proliferation after miR-1287-5p overexpression

**Fig. 11:**
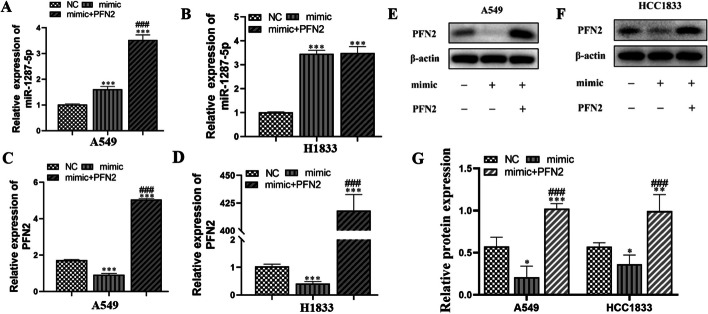
The regulation relationship among miR-1287-5p and PFN2. **A**–**D** RT-qPCR detection showing the expression of miR-1287-5p and PFN2 in both A549 and H1833. Data shown as mean ± SD. ***P < 0.001 versus NC. ^###^*P* < 0.001 versus mimic. **E**–**G** Western blot detection showing the expression of PFN2 in both A549 and H1833. Data shown as mean ± SD. *P < 0.05, **P < 0.01, ***P < 0.001 versus NC. ^###^*P* < 0.001 versus mimic

The RT-qPCR data showed that miR-1287-5p overexpression promoted miR-1287-5p expression in both A549 and H1833 cells after transfection with a miR-1287-5p mimic (Fig. 11A, B). However, miR-1287-5p overexpression decreased PFN2 expression. Transfection with the PFN2-overexpression vector restored miR-1287-5p overexpression at both the protein and mRNA levels (Fig. 11C–G), suggesting that PFN2 is a downstream target of miR-1287-5p.

**Fig. 12:**
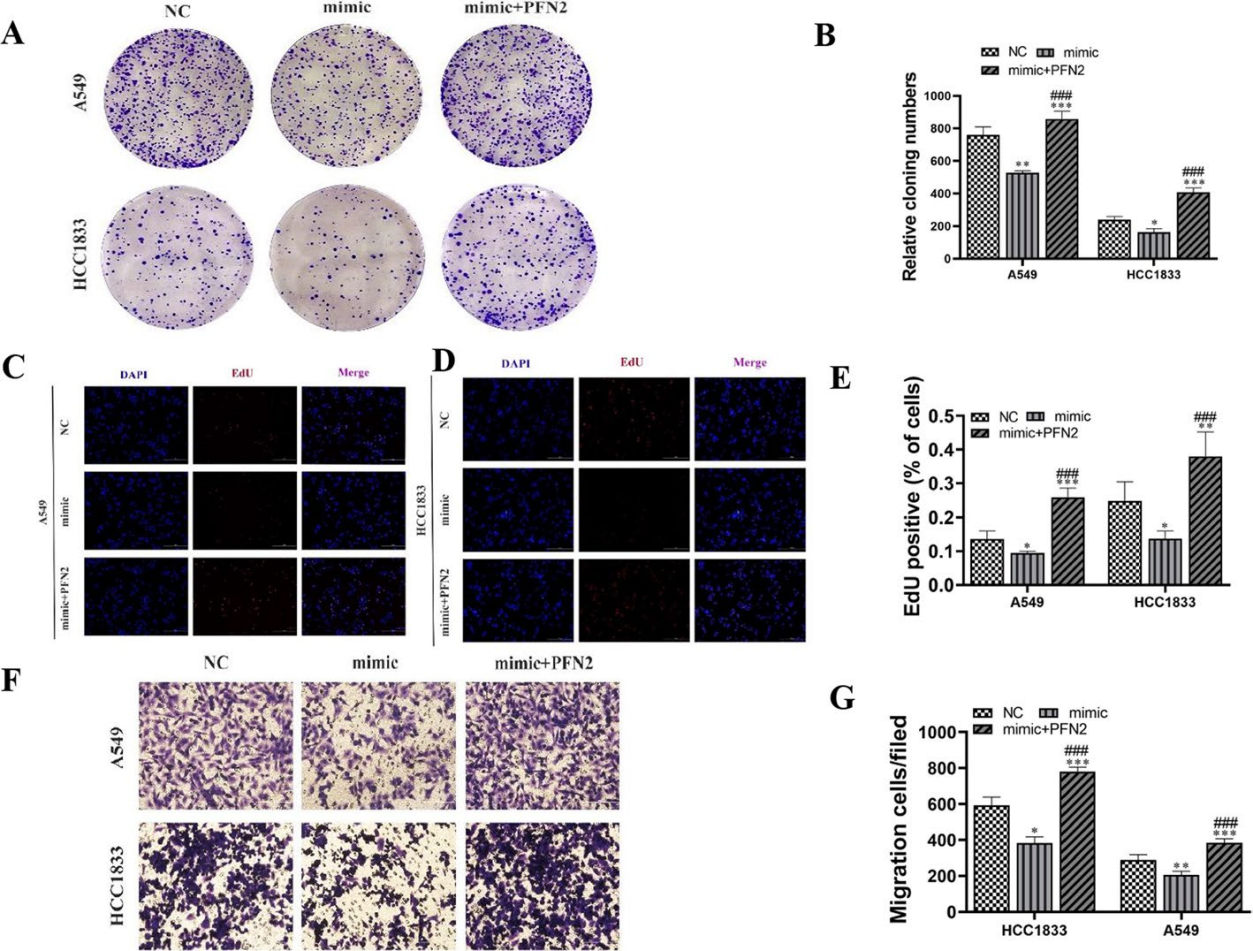
Overexpression of PFN2 reversed NSCLC cell proliferation and migration after miR-1287-5p overexpression. **A**, **B** Clone formation showing the proliferation ability in both A549 and H1833 cells. Data shown as mean ± SD. *P < 0.05, **P < 0.01, ***P < 0.001 versus NC. ^###^*P* < 0.001 versus mimic. **C**–**E** EdU detection showing the proliferation ability in both A549 and H1833 cells. Data shown as mean ± SD. *P < 0.05, **P < 0.01, ***P < 0.001 versus NC. ^###^*P* < 0.001 versus mimic. **F**, **G** Transwell detection showing invasion and migration of A549 and H1833 cells. Data shown as mean ± SD. *P < 0.05, **P < 0.01, ***P < 0.001 versus NC. ^###^*P* < 0.001 versus mimic

The results of the clone formation (Fig. 12A, B) and EdU (Fig. 12C–E) assays showed that overexpression of PFN2 restored proliferation of H1833 and A549 cells following miR-1287-5p overexpression. The results of the transwell assay confirmed that overexpression of PFN2 restored migration of H1833 and A549 cells after miR-1287-5p overexpression (Fig. 12F, G).

## Discussion

Accumulating evidence suggests that circRNAs are important for progression of NSCLC [12]. In the present study and for the first time, hsa_circ_0013561 (circ-SLC16A1) was investigated in tissues from patients with NSCLC, and circ-SLC16A1 expression levels were demonstrated to be significantly upregulated in NSCLC tissues and cell lines. Previous studies have confirmed that SLC16A1 is expressed in almost all human tissues and overexpressed in many cancers, indicating poor prognosis [13–15]. Also, circ-SLC16A1 was mainly located in the cytoplasm, similar to other circRNAs [16]. Clinical studies have confirmed that higher expression of circ-SLC16A1 is negatively correlated with survival and positively correlated with lymphatic metastasis and TNM classification, suggesting important roles in progression of NSCLC.

As a possible regulatory mechanism of circ-SLC16A1, siRNA targeting of circ-SLC16A1 promoted apoptosis and arrested NSCLC cells in the G1 phase. Also, silencing of circ-SLC16A1 inhibited proliferation of NSCLC cells and tumor growth, as well as migration and metastasis of NSCLC cells in lung and lymphatic tissues. Lymphangiogenesis is important for progression and metastasis of many cancers, including NSCLC [17, 18].

Notably, circRNA acts as a prognostic biomarker and inhibits tumor progression through the mechanism of sponge miRNA. The results of the present study revealed that circ-SLC16A1 interacts with miR-1287-5p. Former investigations verified that miR-1287-5p is significantly downregulated in cancers of the lung, cervix, and bone [19–22], suggesting that miR-1287-5p acts as a tumor suppressor. The current investigation discovered that silencing of circ-SLC16A1 promoted expression of miR-1287-5p, while inhibition of miR-1287-5p restored migration and proliferation of NSCLC cells after silencing of circ-SLC16A1. Furthermore, overexpression of miR-1287-5p inhibited migration and proliferation of NSCLC cells, suggesting that circ-SLC16A1 enhances progression of NSCLC via sponging of miR-1287-5p.

Previous studies have confirmed that miR-1287-5p interacts with the 3′-UTR of PFN2 and enhances migration, invasion, proliferation, and epithelial-to-mesenchymal transition of triple-negative breast cancer cells [23]. PFN2 participates in cell shape alternations and cell movement via regulating polymerization and reorganization of the actin cytoskeleton. PFN2 acts as an oncogene in NSCLC [24–26]. In the present study, silencing of circ-SLC16A1 reduced expression of PFN2. Overexpression of PFN2 restored proliferation and migration of NSCLC cells after silencing of circ-SLC16A1 or overexpression of miR-1287-5p. Collectively, these findings suggest that circ-SLC16A1 promotes progression of NSCLC through sponging of miR-1287-5p and increased expression of PFN2.

## Conclusions

The present study demonstrated that circ-SLC16A1 promoted proliferation, migration, and invasion by regulation of miR-1287-5p/PFN2 signaling in NSCLC. Downregulation of circ-SLC16A1 reduced invasion and proliferation of NSCLC cells. Thus, we revealed that circ-SLC16A1 levels are a crucial factor affecting malignant progression in NSCLC patients. These findings suggest that circ-SLC16A1 may serve as a promising diagnostic biomarker for NSCLC.

## Data Availability

All data in this study are available.

## Abbreviations

circRNAs: Circular RNAs
NSCLC: Non-small cell lung cancer
SLC16A1: Solute carrier family 16 member 1
NGS: Next-generation sequencing
CCK-8: Cell counting kit-8
NC: Negative control
CD31: Cluster of differentiation 31
